# Impact of Sex and Age on the Mevalonate Pathway in the Brain: A Focus on Effects Induced by Maternal Exposure to Exogenous Compounds

**DOI:** 10.3390/metabo10080304

**Published:** 2020-07-25

**Authors:** Claudia Tonini, Marco Segatto, Valentina Pallottini

**Affiliations:** 1 Department of Science, Roma Tre University, Viale Marconi 446, 00146 Rome, Italy; claudia.tonini@uniroma3.it; 2 Department of Bioscience and Territory, University of Molise, 86090 Pesche (IS), Italy; marco.segatto@unimol.it

**Keywords:** ageing, brain, cholesterol, mevalonate pathway, sex

## Abstract

The mevalonate pathway produces cholesterol and other compounds crucial for numerous cellular processes. It is well known that age and sex modulate this pathway in the liver. Recently, similar effects were also noted in different brain areas, suggesting that alterations of the mevalonate pathway are at the root of marked sex-specific disparities in some neurodevelopmental disorders related to disturbed cholesterol homeostasis. Here, we show how the mevalonate pathway is modulated in a sex-, age- and region-specific manner, and how maternal exposure to exogenous compounds can disturb the regulation of this pathway in the brain, possibly inducing functional alterations.

## 1. Introduction

**Figure 1:**
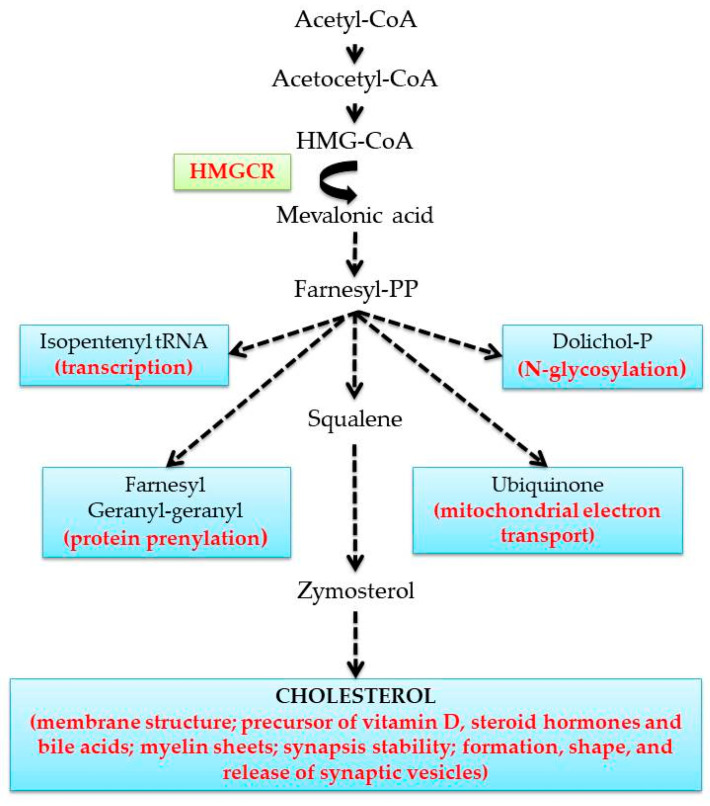
Schematic representation of the mevalonate (MVA) pathway and its end-product functions.

The mevalonate (MVA) pathway produces cholesterol, one of the most important molecules for cellular, tissue, and organism physiology given its crucial structural and metabolic functions. Besides cholesterol, isopentenyl tRNAs, dolichol phosphate, farnesyls, geranylgeranyls, and ubiquinone are also produced by the MVA pathway, and these components are crucial for numerous cellular processes such as transcription, protein N-glycosylation, protein prenylation, and mitochondrial electron transport (Figure 1) [1].

Cholesterol is one of the main components of the plasma membrane determining its chemical-physical properties, such as fluidity and stability. Notably, cholesterol is not uniformly distributed in cell membranes, rather it is concentrated in specialized sphingolipid-rich domains called rafts and caveolae, which are involved in signaling across membranes and thus, are important for cellular functions [2,3]. In the adult brain, about 70–80% of cholesterol is present in myelin sheaths made by oligodendrocytes to insulate axons allowing saltatory electrical signal conduction. Moreover, cholesterol is a precursor for steroid hormones and bile acids [4]. Consequently, imbalanced cholesterol metabolism very often causes pathological changes. For instance, it is well-known that cholesterol accumulation at the artery wall is determinant for the pathogenesis of atherosclerosis and cardiovascular diseases (CVDs). On the other hand, inadequate cholesterol production can likewise be fatal. The suppression of cholesterol biosynthesis in neuronal precursor cells during development results in a reduction of brain size and perinatal lethality in rodents [5].

To maintain proper cholesterol levels, the body employs a large protein network operating in cellular and blood compartments. Cholesterol in human body can both be synthesized by cells and obtained by food intake. Although cholesterol synthesis occurs in all tissues, the liver represents the center of cholesterol homeostasis: it contributes a large fraction to the bodily cholesterol pool, and it helps to eliminate cholesterol by uptake of lipoproteins, storage of esterified cholesterol and its release after conversion into bile acids. Cholesterol synthesis is a complex process that starts with the conversion of acetyl-CoA to 3-hydroxy-3-methylglutaryl-CoA (HMG-CoA). Then, HMG-CoA is converted to mevalonic acid (MVA) by the 3-hydroxy-3-methylglutaryl Coenzyme A reductase (HMGCR), which represents the rate-limiting enzyme in cholesterol biosynthesis. Subsequently, a series of enzymatic reactions leads to the production of 3-isopenenyl pyrophosphate, farnesyl pyrophosphate, squalene, and lanosterol. Finally, a long 19-step process is needed to obtain cholesterol [6]. The cellular level of cholesterol is regulated by an efficient feedback mechanism balancing biosynthesis, import and excretion based on a family of transcription factors known as sterol regulatory element-binding proteins (SREBPs). In sterol-deprived cells, SREBPs precursors are proteolytically cleaved to originate the N-terminal active fragment (n-SREBP), which translocates into the nucleus and activates the transcription of genes required for cholesterol synthesis and uptake [7]. In addition to long-term regulation, HMGCR also undergoes phosphorylation/dephosphorylation, which affect its enzyme activity at a shorter time scale [6]. A plethora of experimental findings demonstrate that peripheral cholesterol homeostasis is sex- and age-dependent, and this peculiarity may be related to the sex-related incidence of cholesterol-dependent pathologies, e.g., CVD [8].

The blood–brain barrier (BBB) separates brain cholesterol from the rest of the body; thus, the homeostatic control of this compound in the central nervous system is independent from the periphery, but probably governed by the same regulatory circuits. Our research group, and other laboratories, recently highlighted that sex and aging can severely influence cholesterol metabolism also in the brain [9,10,11,12,13,14,15].

Here, we will illustrate the sex- and age-dependent differences in cholesterol homeostasis, focusing on the intergenerational effects induced by exogenous compounds in the brain.

## 2. Sex- and Age-Dependent Differences of MVA Pathway in the Liver

A critical problem associated with aging is the increased occurrence of hypercholesterolemia, which represents an alarming risk factor for CVDs. CVDs display dimorphic features that may depend on sex-dependent regulation of cholesterol homeostasis [16]. It has been observed that the flow through the MVA pathway, and in turn cholesterol biosynthesis, is affected by sex and aging. For instance, hepatic HMGCR content and activity are similar in female and male rats at 8 days of age, whereas they develop sexually distinct features at 15-days and 3-months of age. These differences are due to the elevation of plasma estrogen levels, starting from 15 post-natal days in female rats [17]. However, the lower HMGCR activity in female rats does not lead to a concurrent reduction in plasma cholesterol. This discrepancy is explained by the fact that estrogens balance the suppression of cholesterol biosynthesis by increasing intestinal cholesterol absorption [4,18,19]. The dimorphism in MVA pathway regulation is also present during aging. In the elderly, loss of homeostasis frequently leads to changes in the biochemical composition of the body, and hypercholesterolemia represents one of the most common metabolic alterations occurring with increasing age in humans and pre-clinical experimental models [20,21,22]. Although cholesterol plasma levels tend to increase in both males and females with age, the molecular mechanisms underlying the age-related hypercholesterolemia is different between sexes. In males, the age-dependent buildup of reactive oxygen species (ROS) induces hyperactivation of HMGCR, which reflects the increment of cholesterol biosynthesis. Conversely, the fall in plasma estrogen concentration upregulates HMGCR activity, and induces subsequent hypercholesterolemia in females. Notably, both aged male and female rats show decreased HMGCR phosphorylation causing increased cholesterol synthesis. The causes differ: in aged male rats this depends on an ROS-induced hyperactivation of protein phosphatase 2 A (PP2A), while in aged female rats it depends on an estrogen-induced reduction of AMP activated kinase (AMPK) activation. In fact, the activity of AMPK is constant in aged males, but decreases in females [6]. This work has been carried out using separately male and female, but other papers support these sex- and age-dependent results. In fact, it has been demonstrated that age and sex differently impact on cholesterol metabolism in LDL^-/-^ mice [23] and in human beings [24].

Due to its pivotal role in cholesterol biosynthesis, HMGCR is an important pharmacological target for the treatment of hypercholesterolemia. Up to now, the experimental evidence obtained on males has been directly translated to females with respect to clinical practice without considering these well-established sex-differences. In the era of personalized medicine, it seems urgent to consider the age- and the sex-dependent differences to optimize preventive, diagnostic, and therapeutic approaches to combat hypercholesterolemia [8].

## 3. Cholesterol Metabolism in the Brain: What about Sex and Age?

The importance of cholesterol in the central nervous system (CNS) is primarily underlined by its abundance. The brain represents only the 2% of the total body weight but contains 23% of the whole-body cholesterol [25].

Cholesterol is an essential structural component of myelin sheaths and neuronal membranes. The formation, shape, and release of synaptic vesicles, which have particularly high cholesterol content (40 mol%), depend on this molecule [26]. Cholesterol is not equally distributed in membranes of brain cells, instead it is more present in the inner part of the plasma membrane and in the lipid rafts, which regulates a number of molecular processes involved in chemical synaptic transmission [2].

As mentioned above, brain cholesterol metabolism is separated from the rest of the body, since the BBB prevents the passage of lipoproteins. Thus, all cholesterol present in the CNS is synthesized in situ through the MVA pathway [27]. In the mouse brain, cholesterol is synthesized at a rate of 0.26 mg/day during the first week of life. In adult animals, the synthesis exceeds the need and surplus cholesterol is excreted into the plasma at a rate of about 0.023 mg/day [28]. The conversion of cholesterol to 24-S hydroxycholesterol (24S-OHC) represents the major pathway for cholesterol excretion in the brain. Indeed, 24S-OHC is sufficiently hydrophilic to cross the BBB and flow into the bloodstream [29]. The 24S-OHC production is catalyzed by cholesterol 24-hydroxylase (CYP46A1), a cytochrome p450 family member that is mainly expressed in neurons. In addition to this pathway [30], other studies demonstrate that 5α-hydroxy-6-oxocholesterol (3β,5α-dihydroxycholestan-6-one), 7β-hydroxycholesterol and 7-oxocholesterol, which are generally considered cholesterol metabolites formed through reactive oxygen species, can contribute to cholesterol removal from the brain at rates of about 0.1, 2, and 2 mg/24 h, respectively [31].

**Figure 2:**
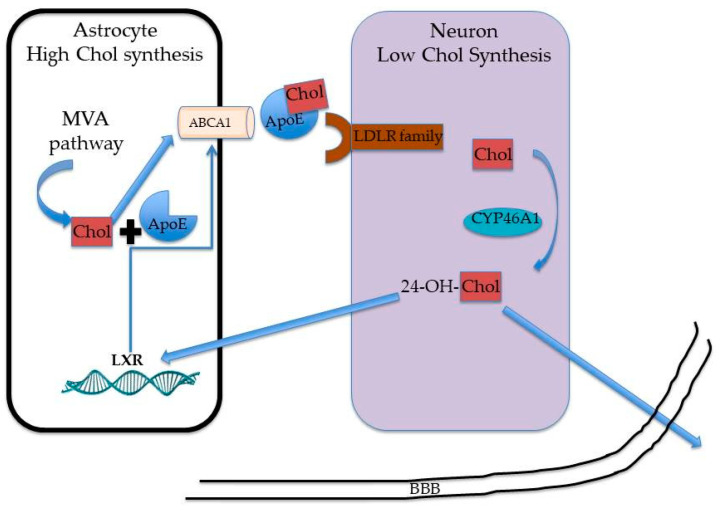
Schematic representation of cholesterol interplay between astrocytes and neurons.

A well-accepted model for cholesterol homeostasis in the brain suggests that during the embryonic stage, the period of major growth and cholesterol-rich myelin formation, and before astrocyte differentiation, neurons are able to meet their need for cholesterol by biosynthesis. Postnatally, neurons are thought to attenuate their synthesis, and import cholesterol from astrocytes. Indeed, cholesterol biosynthesis in glial cells is maintained at high rates also in the adult brain. Once synthetized, cholesterol is integrated into apolipoprotein E (apoE)-containing lipoproteins, which are secreted by astrocytes through ATP Binding Cassette A1 (ABCA1) [32,33]. The transcription factor coding for ABCA1 is the nuclear receptors liver X receptor (LXR), which is activated by 24S-OHC (Figure 2) [34]. Subsequently, apoE-containing lipoproteins are then taken up by neurons through endocytosis mediated by Low Density Lipoprotein Receptor family members (LDLR, LRP1, LRP1b, LRP2, VLDLR) [35]. Therefore, the import of cholesterol from astrocytes may allow neurons to save energy for the generation of electrical activity [36].

The MVA pathway is differently regulated in brain regions, which are known to differ in energy balance, metabolism, cytoarchitecture, and white matter composition. HMGCR exhibits specific expression and activation profiles among different brain areas. For instance, cortex, hippocampus, brain stem and cerebellum are characterized by different protein levels of the enzyme. In particular, HMGCR content is high in hippocampus and cortex, and very low in brain stem. Furthermore, HMGCR is more phosphorylated in brain stem than in hippocampus, cortex, or cerebellum, corroborating a region-specific activation of the MVA pathway [10]. Similarly, sterol regulatory element binding protein 2 (SREBP2) and other proteins involved in the regulatory network show distinct distribution and activation patterns in the brain [10,37]. In addition, regional distribution of LDLR [10] and lipolysis-stimulated lipoprotein receptor (LSR) [38], two important receptors involved in cholesterol-rich lipoprotein uptake, has been reported as well. High activation of the MVA pathway does not necessarily correspond to a high content of cholesterol, or vice versa. In fact, even though the brain stem possesses the highest cholesterol content with respect to other brain areas [39,40], the activity of MVA pathway appears nearly suppressed [10]. These data agree with other reports demonstrating a different cholesterol turnover in distinct brain regions [41,42]. The reported evidence emphasizes that the different levels of key proteins controlling cholesterol metabolism across the brain may reflect the regional needs of cholesterol required for proper functioning [43,44].

The MVA pathway shows not only regional, but also age- and sex-specific differences. In fact, it has been demonstrated that HMGCR levels are lower in the hippocampus of 3-month-old female rats than in age-matched males. Moreover, differences in LDLR were also observed in aged rats, since its expression is higher in the hippocampus and lower in the cortex of females with respect to age-matched males [11]. This sex- and age-dependent dimorphism, especially observed in regions crucial for learning and memory, may have clinical relevance, as marked disparities in the incidence, manifestation, prognosis, and treatment of neurodegenerative disease have been observed between the sexes.

Mutations in genes involved in the MVA pathway or cholesterol metabolism, cause neurologic and psychiatric diseases such as Smith–Lemli–Opitz syndrome (SLOS), Niemann-Pick type C disease (NPC), and desmosterolosis [26]. However, other brain diseases have been related to MVA/cholesterol metabolism such as Autism spectrum disorder (ASD) [2], Huntington’s disease (HD) [29,45], Alzheimer’s disease (AD) [27,46,47,48], and Parkinson disease (PD) [49]. Interestingly, most of them display sex-related differences either on the incidence or severity of symptoms, such as NPC [50,51], AD [52,53,54], ASD [55,56,57], PD and HD [58]. In this context, more efforts are required to clarify whether the sex-dependent disparities are due to differences in MVA/cholesterol metabolism.

## 4. Modulation of MVA Pathway by Endogenous and Exogenous Compounds

The MVA pathway, and in particular HMGCR, are regulated by endogenous signals to maintain the proper cholesterol content. The principal regulators are the major end-products of the biosynthetic pathway itself, which act through negative feedback mechanisms. Notably, specific proteins can monitor the intracellular level of sterols by means of a polytopic intra-membrane sequence called Sterol Sensing Domain (SSD) [4,28]. Moreover, the MVA pathway, namely HMGCR activity, is tightly controlled by several hormonal signals under physiological conditions. These include insulin, glucagon, glucocorticoids, thyroid hormones, and estrogen. Insulin appears to stimulate HMGCR activity by increasing its transcription rate by promoting SREBP-1 and SREBP-2 activity, leading to increased synthesis of both fatty acid and cholesterol in liver and extra-hepatic tissues [59,60]. On the contrary, glucagon reduces plasma cholesterol content [61], mainly by increasing the level of hepatic LDLR [62,63]. HMGCR is also controlled by the circadian rhythm due to variation of the levels of insulin and glucagon. Thyroid hormones promote cholesterol synthesis inducing *hmgcr* gene transcription and enhancing mRNA stability [64]. Moreover, thyroid hormones control HMGCR activity decreasing phosphorylation via AMPK [65]. Finally, glucocorticoids decrease HMGCR protein levels [61]. Regarding estrogens, conflicting data are reported. Experimental evidence indicated that estrogens increase hepatic HMGCR activity by stabilizing its transcript levels [61]; nevertheless, 17β-estradiol also decreases HMGCR levels via feedback regulation following the increased cholesterol uptake [66,67].

Aside endogenous signals, the MVA pathway can also be regulated by exogenous compounds. The most well-known are statins, a class of molecules that lowers cholesterol biosynthesis by irreversible inhibition of HMGCR activity. In 1976, Endo and coworkers isolated the first natural HMGCR inhibitor (mevastatin, also known as compactin) from *Penicillium citrinum* [68]. Four years later, lovastatin (or mevinolin) was isolated from *Aspergillus terreus* [69]. These natural statins are produced via polyketide pathways. Specifically, polyketides constitute a large group of structurally different secondary metabolites synthetized by fungi. To date, the reason why fungi produce HMGCR inhibitors is not entire clear. These compounds may inhibit the growth of environmental competitors [70]. Considering their powerful inhibition of HMGCR activity and, in turn, cholesterol biosynthesis, natural and synthetic statins successfully entered clinical practice, becoming the gold standard to reduce hypercholesterolemia and the risk of CVD [71,72]. Despite the extensive clinical use of statins, evidence for putative sex-dependent differences is inconclusive and limited. For instance, statins seem to induce similar effects between the sexes in secondary CVD prevention, but conflicting data are reported concerning primary CVD prevention [73]. Two main causes may be at the root of these conflicting data: (i) the poor representation of women in clinical trials; (ii) the fact that women adhere less to the treatment because of family caregiving and more severe adverse side effects [74].

Different experimental models have shown that the MVA pathway can be modulated by several exogenous compounds other than statins, such as particulate matter (PM 2.5) [75], Bisphenol A (BPA) [76], polyprenols [77], Omega3 fatty acids [23,78,79], antioxidants [80,81], tocotrienols [82], and myclobutanil [83]. Unfortunately, most of the studies analyzed the effects of exogenous compounds without addressing sex-dependency and thus missing a critical factor in understanding the impact of these compounds on the MVA pathway. Just to give an example, a recent study demonstrates that female mice are more susceptible than their male counterparts to ambient PM2.5 exposure, with cholesterol levels increased only in exposed female mice compared to control group [84]. Furthermore, evaluating the toxicity of the fungicide myclobutanil (MYC) in zebrafish, Pang and colleagues demonstrated a marked sex-specific modulation of liver cholesterol metabolism. In particular, the authors found that exposure to MYC increases the levels of genes involved in the cholesterol synthesis, including HMGCR, in female animals, while they observed an opposite effect in males, where expression levels were significantly reduced [83].

### Maternal Exposure Effects of Exogenous Compounds on MVA Pathway in the Brain

It is well-established that the exposure to pollutants, drugs and other exogenous compounds during pregnancy and lactation represent a serious health concern not only during fetal and postnatal development, but also during adulthood. The hypothesis of a fetal origin of adult diseases states that any challenge occurring in utero permanently changes the body’s structure and function in ways which program the appearance of disease in later life’ [85]. To date, numerous papers demonstrate that maternal exposure to exogenous and potentially dangerous compounds causes disorders in the offspring [86,87,88,89,90].

Any compound impacting HMGCR activity, if able to cross the BBB, can affect the activation of MVA pathway in the brain, thus inducing prospective functional alterations, which can also be related to altered behavior [28,46,91]. A critical function of MVA pathway in the brain is the regulation of neurite elongation [92,93]. In particular, the rate of neurite extension increases upon MVA pathway inhibition, suggesting that an abnormal activity of this pathway during pregnancy and/or lactation may negatively impact this critical period of brain development. Moreover, changes in cholesterol production can affect neurotransmission by altering synapse structure, formation, and plasticity [32,46]. Perinatal inhibition of the MVA pathway by simvastatin prevents the detrimental effects on affective and cognitive components induced by a high fat diet in the offspring [94] supporting the idea that a proper amount of cholesterol is crucial for brain function.

Some drugs, commonly used to treat neurological disorders, and often prescribed to pregnant women, may have profound effects on brain development in the offspring. For instance, maternal exposure to aripiprazole (ARI), used to treat patients with schizophrenia and bipolar disorders, inhibits the 7-dehydrocholesterol reductase (DHCR7), the last enzyme in cholesterol biosynthesis. The inhibition causes accumulation of 7-dehydrocholesterol (7-DHC) in the brain of embryos [95] and affects neuronal viability, proliferation and differentiation. Notably, mutation in the gene encoding DHCR7 is causative for Smith–Lemli–Opitz Syndrome (SLOS), a neurodevelopmental disorder characterized by multiple congenital malformations in different organs, intellectual disabilities, and behaviors characteristic of autism spectrum disorders [27], unfortunately in this study the sex-dependent difference have not taken in consideration. We have shown that maternal exposure to valproic acid (VPA), a drug used to cure epilepsy, induces sex-, age-, and region-specific alterations of the MVA pathway in the offspring’s brains. In particular, cerebellum, cortex, hippocampus, and nucleus accumbens were affected in a sex-dependent manner, whereas no changes were shown in amygdala and dorsal striatum. These alterations were inhomogeneous, leading to hyperactivation or suppression of MVA pathway in relation to each brain area and in dependence on age. This peculiar behavior suggests a complex regulation depending on the distinctive structures and functions, and on the sexual dimorphism characterizing this organ. For these reasons, it is not surprising that the response to the same stimulus differ substantially depending on the brain region and the physiological context [96,97]. Prenatal exposure to VPA is a well validated experimental model of ASD [98], suggesting that the VPA-induced effects on MVA pathway may be connected with this neurodevelopmental disorder displaying a sex-specific onset with a 3:1 male to female ratio [55]. Sex- and region-dependent effects have also been reported following in utero ethanol exposure in rodents. For instance, Soscia and colleagues found decreased cholesterol levels in the cerebellum of newborn rats exposed to ethanol during gestation [99]. Similarly, other studies on rat fetuses prenatally exposed to ethanol revealed a reduction in the amount of cholesterol in the neocortex. Western blot analyses suggest that the reduction of cholesterol levels is due to increased cholesterol efflux, as ABCA1 transporters were significantly upregulated upon ethanol prenatal exposure. Importantly, the authors highlighted a sex-dependent effect since cholesterol metabolism was only affected in the brains of female fetuses [100]. Another study suggested that prenatal ethanol exposure exerts long-term effects on the offspring, as adult rats prenatally exposed to ethanol showed an increased brain cholesterol content [101]. Notably, fetal alcohol syndrome (FAS) shares several common features with SLOS, being characterized by growth retardation, facial abnormalities, and behavioral alterations [100]. Considering that morphological and behavioral dysregulations observed in SLOS are caused by mutations in the gene encoding for DHCR7, further research will reveal whether disturbances in cholesterol metabolism contribute to the outcomes associated to FAS. Furthermore, shedding light on these molecular mechanisms may lead to novel therapeutic strategies based on cholesterol modulation.

Several studies suggest effects in the offspring after maternal exposure to plastic pollutants. For instance, phthalates, present in many consumer products, have received both media attention and regulatory scrutiny because of their toxic effects on reproduction and development [102,103]. Xu and colleagues reported that Di-(2-ethylhexyl)-phthalate (DEHP), one of the most widely used industrial plasticizer, exerts detrimental effects on brain lipid profile upon maternal exposure. Indeed, the administration of DEHP at the dose of 1500 mg/kg from the beginning of the rat gestation significantly reduced the sphingomyelin and free cholesterol content of the brain of the offspring [104]. Besides DEHP, bisphenol A (BPA) is often considered a prototype exogenous molecule to study the impact of contaminants on human and environmental health. Exposure to this compound has been associated with serious endocrine-disrupting effects in humans and wildlife [105]. The effects of maternal exposure to BPA are principally obesity and dyslipidemia [106]. More recently, it has been demonstrated that exposure of rats to BPA during gestation and lactation, even at low doses (10 μg/kg/day), induces life-long dimorphic changes in metabolic homeostasis of the offspring: at weaning, female pups have higher plasma cholesterol and triacylglycerol levels than males, while at adult age, males have lower visceral fat than females. Notably, only females show hyperactivity suggesting that BPA can induce sex-dependent changes in behavior [107]. Maternal exposure to BPA may interfere with developmental programs in offspring, producing adverse outcomes, specifically altering the dimorphic development of many neuronal networks [108]. Indeed, most research on BPA focuses on sex-dependent differentiation of brain regions controlling reproduction, estrogen and testosterone signaling [108] and the neuroendocrine system [109,110,111,112,113,114].

**Figure 3:**
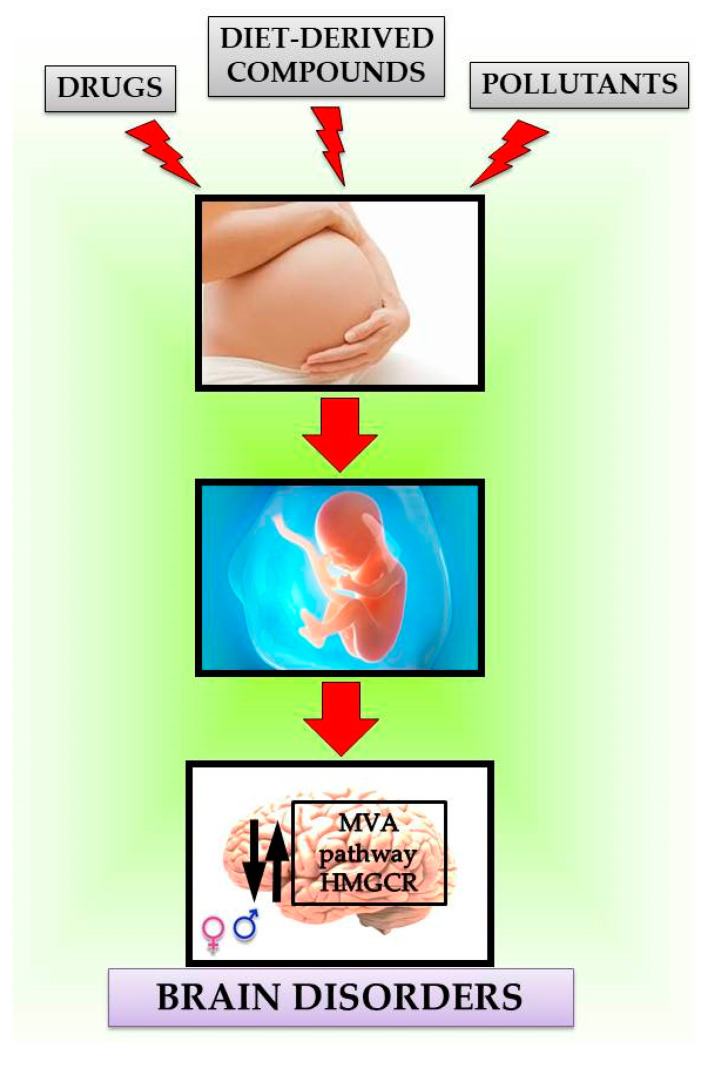
Working model.

Prenatal exposure to BPA mainly affects lipid metabolism, at least in peripheral tissues [115,116,117], but its effects on brain lipid metabolism are not very well understood. Recently, we demonstrated that prenatal exposure to BPA, even at a dose lower than 4 µg/kg/day (the approved threshold by European Food Safety Authority) affects cholesterol metabolism in the brain of rat fetuses [118]. In exposed animals, HMGCR activity was increased. Similarly, LDLR levels were also higher in the BPA group compared to controls, suggesting a significant loss of cholesterol homeostasis. The modulation of HMGCR activity was paralleled by changes in proteins associated with the MVA pathway. In particular, the active fractions of RhoA and Ras, prenylated proteins controlling neurite outgrowth, synaptic connectivity, and memory [46], were affected in the brains of fetuses upon maternal exposure to BPA. Interestingly, BPA only induced sex-dependent alterations at the highest dose tested (250 μg/kg/day), and enhanced HMGCR activity in male, but not in female fetuses. On the contrary, no sex-dependent modulation was observed at the dose of 2.5 μg/kg/day [118]. Since proper activation of the MVA pathway assures appropriate neurite outgrowth [92,93] and its disturbance contributes to neurodevelopmental disorders such as ASD [96,97], these data suggest a dangerous role of in utero BPA exposure in brain development (Figure 3).

## 5. Conclusions and Perspectives

The MVA pathway is crucial for brain development and functioning. The importance of normal sterol metabolism is evidenced by the many genetic disorders associated with mutations in cholesterol biosynthesis enzymes. Prenatal exposure to drugs and chemicals present in the environment, food, and consumer products, can affect key developmental pathways. When exogenous compounds cross the placenta- and blood-brain barrier, they can perturb brain development and cause neuro-pathological changes in the offspring. It is well-accepted that exogenous factors strongly contribute in the etiology of childhood and adult disease. The findings reported here support the hypothesis that deleterious effects of common chemicals and drugs are due, at least in part, to their ability to disrupt MVA pathway. However, further investigations are needed to clarify critical points: the molecular mechanisms mediating long-term effects of exogenous compounds are largely unknown and the specific chemical concentration ranges that pose a risk to health during prenatal development are still elusive. Moreover, data on putative brain epigenetic modulations induced by mother exposure to exogenous compounds and affecting MVA pathway in the brain are missing.

Sex-specific differences in response to external stimuli are present during prenatal life. However, there is a gap in knowledge about the mechanism explaining how sex modulates the susceptibility with respect to environmental chemicals in the offspring. Knowing the sex-dependent responsiveness of the fetus will be indispensable to instruct specific interventions and recommendations.
